# Influence of a garlic-based additive on the performance parameters and intestinal morphology of replacement gilts in Ukraine under commercial conditions

**DOI:** 10.14202/vetworld.2026.1229-1245

**Published:** 2026-03-23

**Authors:** Vadym Lykhach, Natalia Bevz, Anna Lykhach, Ivan Balanchuk, Rostyslav Faustov, Volodymyr Shaposhnik

**Affiliations:** 1 Department of Animal Technology, Faculty of Animal Science and Water Bioresources, National University of Life and Environmental Science of Ukraine, Kyiv, Ukranine; 2 Department of Applied Biology, Animal Breeding and Genetics, Faculty of Animal Science and Water Bioresources, National University of Life and Environmental Sciences of Ukraine, Kyiv, Ukraine; 3 Department of Animal Nutrition and Feed Technology after P. D. Pshenichnyi, Faculty of Animal Science and Water Bioresources, National University of Life and Environmental Sciences of Ukraine, Kyiv, Ukraine; 4 Laboratory of Innovative Technologies and Experimental Livestock Facilities, Institute of Pig Breeding and Agriculture Production, National Academy of Agrarian Sciences of Ukraine, Poltava, Ukraine; 5 Executive Director of the Expert Center for Diagnostics and Laboratory Support, Biolights LLC, Podilska Street, 46, Ternopil, 46003, Ukraine

**Keywords:** antibiotic-free pig production, feed efficiency, garlic phytogenic additive, intestinal histomorphology, phytogenic feed additives, replacement gilts, small intestine morphology, swine nutrition

## Abstract

**Background and Aim:**

The global ban on antibiotic growth promoters (AGPs) in livestock production has increased the search for safe and effective natural alternatives that can maintain productivity and support animal health. Phytogenic feed additives from medicinal plants are gaining more attention because of their antimicrobial, antioxidant, and immunomodulatory properties. Garlic (*Allium sativum*) and caraway (*Carum carvi*) contain bioactive compounds that can enhance digestive function and intestinal health in monogastric animals. Replacement gilts are a critical group in pig production, as their growth and intestinal development during rearing influence their reproductive longevity and herd productivity. However, limited information is available on how phytogenic additives affect intestinal structure and performance in replacement gilts. Therefore, this study aimed to assess the effects of a garlic-based phytogenic additive, “Imunochasnyk,” on growth performance, feed efficiency, survival rate, and small intestine histomorphology of replacement gilts under commercial production conditions.

**Materials and Methods:**

A controlled feeding trial was conducted on 160 clinically healthy crossbred replacement gilts (Large White × Landrace) aged 11–28 weeks under commercial conditions in southern Ukraine. Animals were randomly assigned to two groups: a control group receiving a basal diet (BD) and an experimental group receiving BD supplemented with 0.10% phytogenic additive “Imunochasnyk,” which contains *A. sativum* and *C. carvi* (1000 g/ton). Each group included 80 animals. Growth performance parameters, including live weight, average daily gain (ADG), feed conversion ratio (FCR), and survival rate, were monitored throughout the experimental period. At 190 days of age, five representative gilts from each group were selected for histological examination of the jejunum. Morphometric analyses of enterocyte dimensions, nucleus-to-cytoplasm (N:C) ratio, and structural characteristics of villi, crypts, goblet cells, and Paneth cells were performed. Data were expressed as mean ± standard error of the mean and analyzed statistically, with significance set at p < 0.05.

**Results:**

Dietary supplementation with the phytogenic additive significantly enhanced the productive performance of replacement gilts. Starting from week 17, the experimental group showed greater live weight and higher ADG than the control group. At 28 weeks of age, the final live weight reached 128.52 kg in the supplemented group compared to 118.64 kg in the control group (p < 0.001). The FCR decreased by 8%–11% during intensive growth phases, indicating improved feed utilization efficiency. The survival rate increased to 95.0% in the supplemented group compared to 85.0% in the control group. Histological analysis revealed notable intestinal adaptations in supplemented gilts, including a 19.6% increase in enterocyte area and a 31.0% increase in nuclear area, leading to a higher N:C ratio (p < 0.01). Additionally, increased villus tortuosity, enlarged crypt lumina, higher goblet cell density, and activation of Paneth cells were observed, suggesting enhanced absorptive capacity and strengthened mucosal defense mechanisms.

**Conclusion:**

Including the garlic-based phytogenic additive “Imunochasnyk” at 0.10% in the diet significantly enhanced growth performance, feed efficiency, and intestinal structure in replacement gilts. The additive stimulated epithelial metabolic activity, boosted mucosal defenses, and improved nutrient absorption, ultimately increasing productivity and survivability. These results support the use of garlic-based phytogenic additives as sustainable alternatives to antibiotics in intensive pig production systems.

## INTRODUCTION

Enhancing the efficiency of replacement gilt rearing remains a key focus in modern global pig production. Animals are constantly exposed to various stressors in intensive farming systems, including technological transitions, dietary changes, and high pathogen pressure. Sub-therapeutic doses of antibiotics were traditionally used to address these challenges. However, the increasing global concern over antimicrobial resistance and subsequent legislative bans on antibiotic growth promoters (AGPs) in the European Union and several Asian countries have driven an urgent search for safe and effective bio-alternatives. Plant-derived feed additives, commonly called phytogenic feed additives or phytobiotics, have gained particular attention because of their multi-targeted biological activities [1, 2].

Phytobiotics, especially those derived from phytoextracts and essential oil blends of garlic (*Allium sativum*) and caraway (*Carum carvi*), are recognized as powerful natural growth stimulants and modulators of the gastrointestinal environment. Garlic contains a wide range of bioactive organosulfur compounds, notably allicin, alliin, and diallyl sulfides, which show strong antimicrobial, antioxidant, and anti-inflammatory effects [3–5]. Caraway is rich in carvone and limonene, compounds known for their antispasmodic effects and their ability to promote the secretion of endogenous digestive enzymes [6, 7]. The synergistic combination of these components requires a comprehensive assessment of their effects, moving beyond simple performance measures such as live weight, average daily gain (ADG), and survival rate toward a more detailed analysis of the morphofunctional parameters of the digestive tract [4, 8].

Recent systematic reviews and large-scale trials [9–12] have confirmed that phytogenic additives generally stabilize the structure of the intestinal microbiota and reduce the metabolic costs of maintaining the gut mucosal barrier. Pandey *et al*. [7] and Wang *et al*. [2] highlighted that these compounds enhance nutrient digestibility by increasing the absorptive surface area of the small intestine. Garlic-derived formulations have been shown to increase villus height and decrease crypt depth in both piglets and poultry, changes that are directly linked to improved feed conversion efficiency [13–17]. However, much of the existing evidence comes from studies on weaned piglets or finishing pigs, leaving a significant knowledge gap about other specialized production categories.

Replacement gilts constitute a unique physiological group that has been surprisingly understudied in pig nutrition research. Unlike finishing pigs, where the main goal is rapid muscle growth and short-term weight gain, rearing replacement gilts emphasizes synchronized body development and long-term reproductive maturity. The period between 11 and 28 weeks of age marks a vital developmental stage. During this time, the health and function of the intestine not only affect current growth rates but also impact the systemic absorption of micronutrients and energy needed for future reproductive success and the transition to the first farrowing [18–22]. Failing to optimize gut health during this phase can lead to premature culling, significantly compromising the economic viability of pig production systems.

Furthermore, most traditional histological studies in swine nutrition focus on gross morphometric indicators like villus height and crypt depth. While informative, these parameters only provide a superficial view of intestinal health. A more detailed assessment using high-resolution cytological endpoints is needed to better understand the metabolic state of the intestinal epithelium. The enterocyte nuclear-to-cytoplasmic (N:C) ratio acts as a sensitive marker of cellular protein synthesis and metabolic activity. Likewise, the activation and density of Paneth cells, which serve as primary defenders of the intestinal crypts, offer direct evidence of the mucosa’s innate secretory defense capacity.

Beyond the mucosal layer, the role of the enteric nervous system (ENS), especially the myenteric (Auerbach’s) plexus, is often overlooked in animal nutrition studies. The ENS controls gastrointestinal motility and local blood flow, and structural changes within this system may be a key mechanism through which phytogenic additives improve nutrient transit and absorption. Combining performance data with these advanced histological markers is therefore crucial to support claims about the probiotic-like or growth-promoting effects of phytogenic formulations under commercial production conditions.

Despite accumulating evidence supporting the beneficial effects of phytogenic feed additives in swine production, several significant knowledge gaps remain. Most existing studies have focused on weaned piglets or finishing pigs, whereas replacement gilts, a physiologically distinct production category with specific developmental and reproductive needs, have received comparatively little scientific attention [23–25]. Additionally, many studies evaluating phytogenic additives from *A. sativum* and *C. carvi* have primarily focused on basic performance measures, such as growth rate, feed efficiency, and carcass traits. Comprehensive assessments that combine productive performance with detailed morphofunctional analyses of the intestinal tract are still rare. Specifically, high-resolution cytological indicators of intestinal activity, such as the N:C ratio, Paneth cell activation, and adaptive changes in the intestinal villi and crypts, have seldom been studied together with productive responses in replacement gilts. Moreover, the potential role of the ENS, especially structural changes in the myenteric (Auerbach’s) plexus that regulate intestinal motility and nutrient transit, has received limited attention in prior swine nutrition research. These gaps emphasize the need for integrated studies that combine productive performance metrics with advanced histological and cytological analyses under commercial farming conditions, to better understand the biological mechanisms behind phytogenic supplementation in replacement gilts.

Therefore, the present study aimed to evaluate the effects of a garlic-based phytogenic feed additive (“Imunochasnyk”), containing bioactive compounds derived from *A. sativum* and *C. carvi*, on the productive performance and intestinal structural and functional characteristics of replacement gilts reared under commercial conditions. Specifically, the study aimed to determine the influence of dietary supplementation with this phytogenic additive on live weight dynamics, ADG, feed efficiency, and survival rate during the rearing period from 11 to 28 weeks of age. Additionally, the study sought to investigate structural and cellular adaptations of the small intestine associated with phytogenic supplementation, including changes in enterocyte morphology, the N:C ratio, villus structure, crypt architecture, and the activity of goblet and Paneth cells. Particular attention was also given to assessing potential alterations in the neuromuscular components of the intestinal wall, especially neurons of the myenteric (Auerbach’s) plexus, as indicators of modified gastrointestinal motility and functional adaptation. By combining productive performance metrics with detailed histological and cytological analyses, this study aimed to provide mechanistic evidence supporting the use of garlic-based phytogenic additives as sustainable alternatives to AGP in modern intensive pig production systems.

## MATERIALS AND METHODS

### Ethical approval

The study was conducted in strict accordance with European animal welfare legislation (Council Directive 2008/120/EC and Directive 2010/63/EU) [26, 27], as well as with national Ukrainian regulations (Order of the Ministry of Economy of Ukraine, February 18, 2021) [28]. The Bioethics Commission of the National University of Life and Environmental Sciences of Ukraine approved the experimental protocol (June 26, 2025; Protocol No. 039). Additionally, the study design and reporting adhered to the Animal Research: Reporting of *In Vivo* Experiments guidelines to promote transparency and reproducibility.

### Study period and location

The study was conducted from March to September 2025 at the Private-Lease Enterprise “Viktoria” located in the Bashtanka district of the Mykolaiv region, Ukraine. The region is characterized by a temperate continental climate with mild winters and hot summers, average annual precipitation of 300–350 mm, and approximately 2116 h of sunshine per year. A total of 160 clinically healthy crossbred replacement gilts (Large White × Landrace; PIC Genetics, UK) were included in the experiment.

The animals’ health status was verified through thorough veterinary screening, which confirmed they showed no signs of respiratory distress, maintained normal body temperatures (38.5°C–39.5°C), and had normal fecal consistency. Only gilts with no history of systemic antibiotic treatment for at least 14 days prior to the trial were included. Replacement gilts were selected as the experimental group because their intestinal health and morphofunctional development during the rearing period are crucial to reproductive longevity and the long-term economic sustainability of breeding herds.

### Group allocation and experimental design

**Figure 1:**
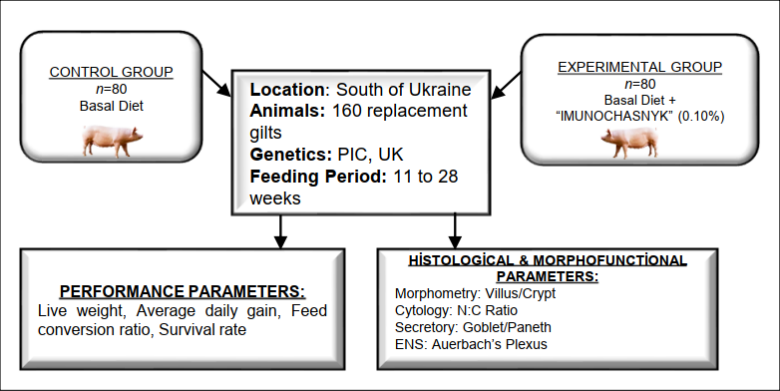
General scheme of the study.

Figure 1 presents the experimental design. At the start of the rearing period (11 weeks old), gilts with an initial live weight of 31.96–32.62 kg were divided into two experimental groups: control (n = 80) and experimental (n = 80). A stratified randomization process was used, employing a computer-generated random number table based on initial body weight to ensure similar conditions at the start. The experimental period ran from 11 to 28 weeks of age and included a 7-day acclimation phase before dietary supplementation began.

The experiment was designed as a controlled feeding trial conducted under commercial production conditions. Although animals were housed in groups of 20 gilts per pen due to technological and social management needs, the individual gilt was considered the experimental unit for all performance and histological measurements. This approach was justified because live weight was monitored individually and intestinal tissue samples were collected from individual animals at the end of the trial. All pens were located within the same facility and managed under identical environmental and husbandry conditions to minimize potential pen effects and ensure comparable physiological status between treatment groups.

### Diets and feed additives

The nutritional program was designed to meet the physiological needs of crossbred replacement gilts during the rearing phase, following the guidelines of the National Research Council [29] and the Pig Improvement Company [30]. All animals received a standard basal diet (BD) formulated as a complete compound feed throughout the study. After the acclimation period (weeks 11–12), the control group continued to receive the BD without any supplementation, while the experimental group received the BD supplemented with the phytogenic additive “Imunochasnyk” at a rate of 1000 g/t (0.10%) from week 12 to week 28.

Feeding was provided ad libitum using specialized diets formulated for replacement gilts and produced at the farm’s feed mill. The amount of the phytogenic additive was based on the manufacturer’s recommendations and previous practical use to ensure an optimal balance between biological effectiveness and feed palatability. Table 1 shows the ingredient composition and the calculated nutritional value of the BD.

**Table 1:** Ingredient composition and calculated nutritional value of the basal diet for replacement gilts.

Item	Unit	Content
Ingredients		
Corn	%	35.0
Barley	%	10.0
Wheat	%	21.0
Wheat bran	%	12.0
Soybean meal (44.5%)*	%	7.00
Sunflower meal (36.0%)*	%	10.0
Cehavit Sow Premix Replacement	%	5.00
Calculated nutritional value		
Metabolizable energy	MJ/kg	12.36
Crude protein	g/kg	155.00
Crude fat	g/kg	24.09
Fiber	g/kg	53.07
Calcium	g/kg	8.08
Total phosphorus	g/kg	5.57
Digestible phosphorus	g/kg	3.05
Sodium	g/kg	2.46
Lysine	g/kg	7.76
Digestible lysine	g/kg	6.27
Methionine	g/kg	3.03
Digestible methionine	g/kg	2.55
Methionine + cystine	g/kg	5.92
Digestible methionine + cystine	g/kg	4.71
Threonine	g/kg	6.16
Digestible threonine	g/kg	4.54
Tryptophan	g/kg	1.75
Digestible tryptophan	g/kg	1.32
Vitamin A	IU/kg	13,000
Vitamin D_3_	IU/kg	2,000
Vitamin E	mg/kg	100.00

* Note: Crude protein content.

The phytogenic additive “Imunochasnyk” (Argano Organis LLC, Ukraine) is a standardized powder formulation containing dried *A. sativum* (95 g/100 g) and *C. carvi* (5 g/100 g). The additive is standardized for essential oil content and sulfur compounds, especially allicin. To preserve the stability of bioactive compounds, it was stored in its original moisture-proof packaging at temperatures below 25°C. Homogeneous mixing into the diet was achieved with a two-step process: the additive was first premixed with 5 kg of BD, then combined with the 1000-kg feed batch using a twin-shaft paddle mixer. Batch uniformity was verified in accordance with the State Standard of Ukraine (4508:2005).

### Housing and management

Housing conditions were arranged following the national standard VNTP-APK–02.05 “Pig Enterprises (complexes, farms, small farms)” and the recommendations of the PIC genetic company [31]. Gilts were housed in groups of 20 animals per pen on concrete slatted floors, with a space allowance of 1.0–1.65 m² per animal. Water was provided *ad libitum* via nipple drinkers, and all veterinary treatments and preventive measures were applied equally to both groups in accordance with the farm’s management protocol.

Environmental conditions within the facility were controlled using a negative-pressure ventilation system consisting of an axial exhaust fan installed in the ceiling and air inlet valves positioned in the side walls. A microprocessor-based climate management system automatically regulated ventilation to maintain microclimatic conditions, including an air temperature of 18°C–20°C, a relative humidity of 40%–60%, and an air velocity of 0.30–1.00 m/s. Manure removal was performed using a vacuum-gravity slurry system with channels beneath the pens and pipelines that transferred manure to external collection tanks. All management procedures, including feeding, watering, housing, and sanitary measures, were carried out in compliance with Ukrainian and European animal welfare legislation, including the Law of Ukraine “On Veterinary Medicine” [28].

### Performance measurements

Productive performance parameters were tracked throughout the experimental period from 11 to 28 weeks of age. Live weight (LW) was individually recorded at weeks 11 (baseline), 12, 17, 22, 26, and 28 using electronic livestock scales (VPD-CK 20) with an accuracy of ±0.1 kg. ADG was calculated for each growth interval and for the entire experimental period using standard formulas [32]. Feed conversion ratio (FCR) was determined for each production phase based on total feed intake recorded per group and the corresponding total weight gain of animals [32, 33]. Daily monitoring of health status, injuries, disease cases, and mortality was conducted, and the survival rate (%) was calculated as the proportion of animals remaining in each group relative to the initial population.

### Histological procedures

At the end of the rearing period (190 days of age), five clinically healthy gilts from each group were selected for histological examination of the small intestine. The limited sample size was chosen due to the high genetic homogeneity and breeding value of replacement gilts, and to comply with ethical principles aimed at minimizing animal use while maintaining adequate statistical sensitivity to detect biologically meaningful differences.

Animals were slaughtered at the farm’s veterinary-sanitary slaughter facility in accordance with animal welfare standards. After a 12 h fasting period, jejunal tissue samples were collected from the middle section of the small intestine, approximately 2 meters distal to the pylorus. Tissue segments roughly 2 centimeters long were excised, rinsed with cold saline, and immediately fixed in Bouin’s solution. Histological processing was carried out at the Laboratory of Histology, Cytology, and Embryology at Petro Mohyla Black Sea National University, following the method developed by Koziy M. S. [34]. Five nonconsecutive sections were analyzed for each animal to ensure representative sampling.

The histological preparation involved fixing in Bouin’s solution for 48 h at 20°C–30°C, dehydrating in 100% diethylene dioxide for 30 minutes with three solution changes, and clearing in ortho-xylene for 20 min at 30°C. Tissue samples were then embedded in a paraffin–lanolin mixture consisting of 85%–90% paraffin and 10%–15% lanolin at 60°C with two changes of embedding medium. Sections were cut using a custom angular microtome and stained with modified Hart’s fuchsin and eosin Y before mounting in Canadian balsam.

Microscopic examination was performed using a Leitz “Diaplan” light microscope (E. Leitz, Wetzlar, Germany) equipped with a Linvatec-2 halogen light source (10–240 W). Microphotographs were taken with a Nikon F-70 camera mounted on a 1.6× binocular adapter and controlled via computerized exposure control. Images were processed using the FS Viewer software package (https://www.faststone.org), and morphometric analysis was carried out with MICAM software (Belgium, http://science4all.nl/?Microscopy_and_Photography). To reduce observer bias, histological slides were coded by an independent researcher, and the observer performed all measurements under a single-blind protocol, unaware of group assignments.

### Morphometric analysis

Morphometric analysis of the prismatic brush-border epithelium was conducted by measuring 50 enterocytes per animal. For each cell, the linear dimensions of both the cell body and nucleus were recorded to calculate the cell area, nuclear area, cytoplasmic area, and the nucleus-to-cytoplasm (N:C) ratio. Additionally, 10 well-oriented villi and 10 intestinal crypts were measured per animal, resulting in at least 50 measurements per specimen. Only villi with a clearly visible central lacteal and intact epithelial coverage from base to apex were included in the analysis.

### Statistical analysis

The individual gilt was used as the experimental unit for all measurements. Before statistical analysis, the normality of data distribution and homogeneity of variance were assessed using Shapiro–Wilk and Levene’s tests, respectively. Statistical analysis was conducted with Statistica 12.0 (StatSoft Inc., 2014). Data are shown as mean ± standard error of the mean (SEM), and differences between groups were considered statistically significant at p < 0.05, with higher significance levels at p < 0.01 and p < 0.001 [35].

## RESULTS

### Growth performance of replacement gilts

A study of the performance parameters of replacement gilts showed that in the early stages of rearing (11–12 weeks), the LW of animals in both groups was nearly identical (32.02–32.62 kg), indicating their homogeneity. No significant differences were observed between the control and experimental groups during this period, and culling due to injury or disease was minimal (0%–2.5%) (Table 2).

**Table 2:** Performance parameters of replacement gilts throughout the rearing period.

Age (weeks)	Parameter	Control (n = 80), mean ± SEM	Experimental (n = 80), mean ± SEM
11	LW (kg)	32.62 ± 0.248	32.02 ± 0.330
12	LW (kg)	36.80 ± 0.434	37.62 ± 0.404
	Culling (injuries and diseases), %	0	2.5
17	LW (kg)	61.40 ± 0.412	66.22 ± 0.384***
	ADG (g)	731.43 ± 4.562	817.14 ± 3.632**
	FCR	2.73	2.45
	Culling (injuries and diseases), %	5.0	2.5
22	LW (kg)	88.16 ± 0.282	95.22 ± 0.314***
	ADG (g)	764.57 ± 4.082	828.57 ± 3.420***
	FCR	3.02	2.79
	Culling (injuries and diseases), %	7.9	0
26	LW (kg)	108.20 ± 0.308	117.80 ± 0.296***
	ADG (g)	715.71 ± 3.502	806.43 ± 2.786***
	FCR	3.42	3.04
	Culling (injuries and diseases), %	0	0
28	LW (kg)	118.64 ± 0.282	128.52 ± 0.226***
	ADG (g)	745.71 ± 4.40	765.71 ± 3.30**
	FCR	3.38	3.29
	Culling (injuries and diseases), %	2.9	0
	Survival rate (%)	85.0	95.0

Notes: Significant:

** p < 0.01;

*** p < 0.001 (in comparison with animals of the control group). SEM = Standard error of the mean.

From the 17th week of rearing, replacement gilts in the experimental group had a significantly higher LW (66.22 kg) than those in the control group (61.40 kg; p < 0.001). Animals supplemented with the feed additive “Imunochasnyk” had significantly higher ADG (817.14 g vs. 731.43 g in the control group, p < 0.01), indicating the additive’s stimulating effect on replacement gilt growth.

At 22 weeks of age, the difference between the groups persisted and even grew: the LW of gilts in the experimental group reached 95.22 kg, which was significantly higher by 7.06 kg (p < 0.001). The ADG during this period was also higher (828.57 vs. 764.57 g, p < 0.001). No culling cases were recorded in the experimental group, while 7.9% of the control group was culled, indicating a positive effect of the additive on the animals’ health and viability.

By 26 weeks of age, the experimental group maintained its lead: the LW of gilts reached 117.80 kg, which was 9.6 kg more than in the control group (p < 0.001). The ADG was also notably higher (806.43 vs. 715.71 g, p < 0.001). No cases of culling were recorded in either group at this stage.

The difference between the groups remained significant at the end of the rearing period (28 weeks). The LW of gilts in the experimental group reached 128.52 kg, surpassing the control value by 9.88 kg (p < 0.001). The ADG also stayed high (765.71 vs. 745.71 g, p < 0.01).

Along with improved growth rates, the experimental group showed better feed efficiency throughout the rearing period. At 17 weeks, the FCR in the experimental group was 2.45, which was 10.3% lower than the control group’s 2.73. A similar pattern appeared at week 26, with the experimental group at 3.04 compared to 3.42 in the control group (an 11.0% reduction). By the end of the trial (week 28), the experimental gilts had a more favorable FCR (3.29) than the control group (3.38), indicating more efficient feed conversion into body mass from the phytogenic additive.

The overall survival rate of animals in the experimental group was 95.0%, compared to 85.0% in the control group, indicating an improvement in physiological condition and a reduction in livestock losses due to the use of the feed additive “Imunochasnyk.”

### Histological structure of the small intestine

A comparative histological analysis of the small intestine was performed to assess the effectiveness of the natural plant-based growth promoter “Imunochasnyk” on the digestive organs of replacement gilts. The microstructural organization of the small intestine in the control group was used as a reference standard.

**Figure 2:**
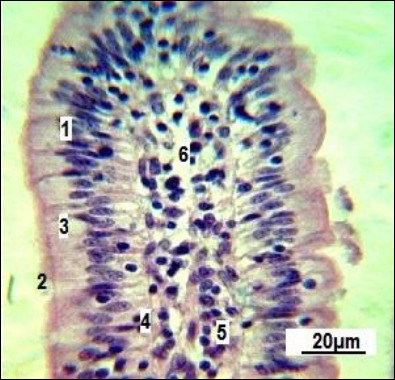
Longitudinal section of a single small intestinal villus in replacement gilts (control group).1: simple columnar epithelium; 2: brush-border; 3: goblet cells; 4: basement membrane; 5: connective tissue stroma; 6: vascular network. Stained with Boehmer’s hematoxylin and modified Hart’s fuchselin. Corrective optical filter ×2.5. Final magnification ×300. Scale bars = 20 μm.

The mucous membrane of the small intestine villi consists of all the structural components of the mucosa. The villi are primarily aligned along the longitudinal axis of the intestine in longitudinal sections. They are covered with a single-layered high prismatic (brush-border) epithelium (Figure 2).

**Figure 3:**
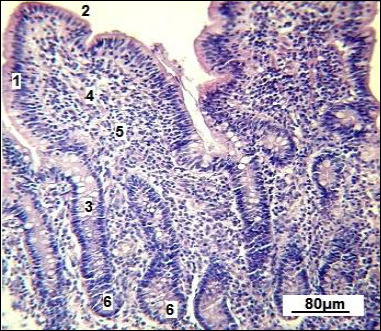
Small intestine villi in replacement gilts (control group). 1: simple columnar epithelium; 2: brush-border; 3: goblet cells; 4: smooth muscle cells; 5: connective tissue stroma and vascular network; 6: Lieberkühn crypts. Stained with Boehmer’s hematoxylin and modified Hart’s fuchselin. Corrective optical filter ×2.5. Final magnification ×120. Scale bars = 80 μm.

Figure 2 shows that goblet enterocytes (3) are occasionally found within the general epithelial layer. Our observations revealed that most of them are located closer to the villi base. The villi stroma is made up of loose connective (5) and reticular tissue. The latter is not clearly differentiated when conventional staining methods are used. Individual smooth muscle cells (4) can be seen within the connective tissue stroma (Figure 3).

**Figure 4:**
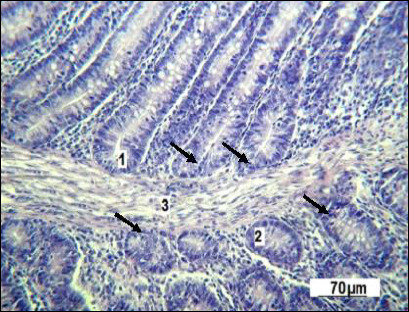
Lieberkühn crypts of the small intestine in the control group. 1: longitudinal section; 2: sagittal section; 3: muscularis mucosae. Stained with Boehmer’s hematoxylin and modified Hart’s fuchselin. Corrective optical filter ×2.5. Final magnification ×140. Scale bars = 70 μm.

The micrograph clearly displays the longitudinal orientation of smooth muscle cells inside the villi. A centrally located, less dense space extending along the long axis of the villus can be seen in several villi, corresponding to the central lymphatic space (central lacteal), which is the initial part of the intestinal lymphatic network. The villus stroma comes from the mucosal lamina propria and contains connective tissue components and blood vessels. Many Lieberkühn crypts are found within the lamina propria, appearing as tubular epithelial invaginations embedded in the connective tissue (Figure 4).

The submucosal layer and the adjacent muscularis mucosae are only partially visible in this section. Some Lieberkühn crypts are seen in both longitudinal and sagittal projections within a single specimen. Given the overall folded structure of the intestine, areas of the lamina propria also contain cross-sections of the tubular glands.

According to Figure 4, the epithelium of the Lieberkühn crypts morphologically matches the cellular composition of the villus surface. Goblet cells in the glands are less common than in the mucosal epithelium of the villi. Also, specific secretory cells with eosinophilic granularity are seen at the base of the glands.

The muscular layer of the mucosa lies directly beneath the bases of the Lieberkühn crypts. The submucosa is quite thick and is made up of loose, irregular connective tissue, Meissner’s plexus ganglia, and many blood and lymphatic vessels.

**Figure 5:**
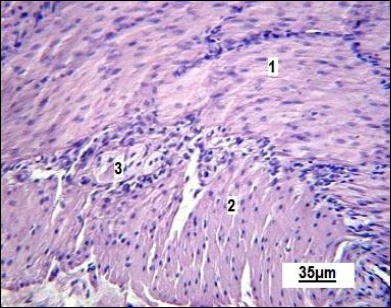
Muscular layer of the small intestine in the control group. 1: circular muscle layer; 2 : longitudinal muscle layer; 3 : Auerbach’s plexus ganglion (myenteric plexus). Stained with Boehmer’s hematoxylin and eosin Y. Final magnification ×300. Scale bars = 35 μm.

The muscular coat of the small intestine consists of two layers. Parasympathetic Auerbach’s plexus ganglion (myenteric plexus) is seen between these layers in the histological sections (Figure 5). In the histological samples, the circular layer appears slightly thicker than the longitudinal one.

**Figure 6:**
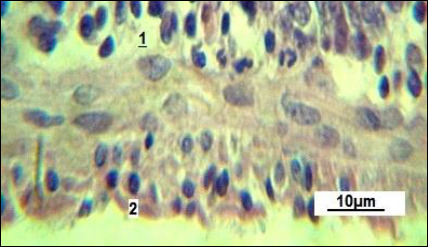
Serous membrane of the small intestine in the control group. 1: connective tissue layer; 2: mesothelium. Stained with Boehmer’s hematoxylin and modified Hart’s fuchselin. Corrective optical filter ×2.5. Oil immersion. Final magnification ×800. Scale bars = 10 μm.

**Figure 7:**
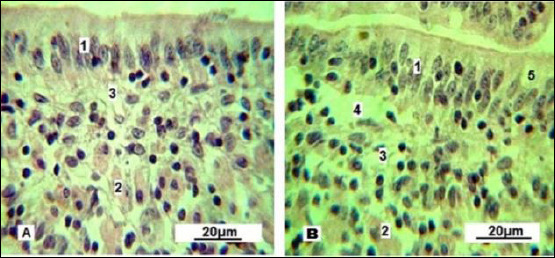
Surface of a small intestinal villus in replacement gilts. 1: brush-border columnar epithelium; 2: cross-sections of smooth muscle cells; 3: loose connective tissue; 4: lymphatic capillary; 5: goblet cells. Stained with Boehmer’s hematoxylin and modified Hart’s fuchselin. Corrective optical filter ×2.5. Final magnification ×600. Scale bars = 20 μm.

The serous membrane consists of a thin layer of connective tissue and mesothelium (Figure 6). The use of the natural plant-based growth promoter “Imunochasnyk” helps improve small intestine absorption, secretion, and peristalsis. Histological analysis showed a variety of morphological changes in the intestinal wall (Figure 7).

As reported in the control group (Figure 7A), the prismatic brush-border epithelium generally maintains stable nuclear polarity, with only occasional displacement of nuclei toward the caudal side. This suggests a normal and consistent pattern of nutrient absorption.

The feed additive “Imunochasnyk” enhanced chyme absorption in the experimental group. Based on the data shown in Figure 7B, some disorganization in nuclear arrangement is evident; most nuclei tend to shift toward the caudal direction. At the same time, there is an increase in apical cell spaces, indicating higher nutrient absorption and their accumulation in the cytoplasm. The nuclei seem displaced toward the basement membrane. Changes in the cytological features of the prismatic brush-border epithelium support this observation.

**Table 3:** Changes in the cytological characteristics of the prismatic brush-border epithelium in replacement gilts, mean ± Standard error of the mean.

Group (n = 5)	Linear dimensions of cells (μm) A × B	Linear dimensions of nuclei (μm) A × B	Cell area (μm²)	Nuclear area (μm²)	Cytoplasm area (μm²)	N:C ratio
Control	7.4 × 5.0 ± 0.95	3.4 × 2.2 ± 0.027	36.98 ± 5.92	7.48 ± 0.29	29.50 ± 6.84	0.25 ± 0.03
Experimental	8.1 × 5.6 ± 1.12	3.5 × 2.8 ± 0.024	44.25 ± 7.43**	9.80 ± 0.35*	34.75 ± 4.86**	0.28 ± 0.04**

Notes: n = number; N:C ratio = nucleus-to-cytoplasm ratio. Fifty cells were analyzed per animal (250 cells per group). Significant:

* p < 0.05,

** p < 0.01 (in comparison with animals of the control group).

The analysis of the data in the table shows that a 1.20-fold increase in cell size corresponds with a 1.31-fold rise in nuclear size. A significant change in the N:C ratio from 0.25 to 0.28 was observed, clearly indicating increased absorptive activity of the epithelial layer.

Specifically, the mean epithelial cell area increased by 19.6% (44.25 ± 7.43 μm² vs. 36.98 ± 5.92 μm² in the control group, p < 0.01). Under these stimulated conditions, changes in the villi’s topography were supported by a higher density of goblet cells, averaging 12–14 cells per villus in the experimental group compared to 8–10 cells per villus in the control group. This demonstrates increased mucin production and strong mucosal surface activation.

**Figure 8:**
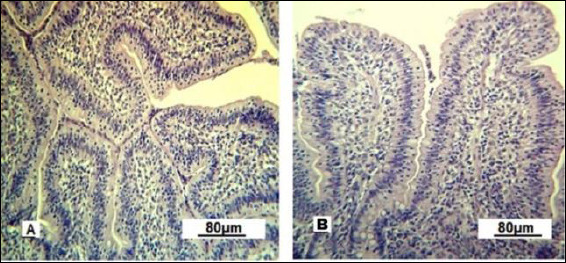
Villi of the small intestine mucous membrane in replacement gilts (experimental group). A: tortuous shape; B: scalloped shape. Stained with Boehmer’s hematoxylin and modified Hart’s fuchsia. Corrective optical filter ×2.5. Final magnification ×120. Scale bars = 80 μm.

In the control group, the villi were short and straight, while under optimized feeding conditions they adopted different shapes (Figure 8). The thickening and tortuosity of the mucosal villi result from increased lymph and blood inflow. The terminal spaces of the lymphatic vessels appeared empty under the microscope in control gilts, whereas they were densely filled with lymphocytes in the intestinal villi of experimental gilts.

The scalloped appearance of villi was seen less often but was found only in the experimental group, indicating an adaptive change in the intestinal mucosa to boost the absorptive surface area.

**Figure 9:**
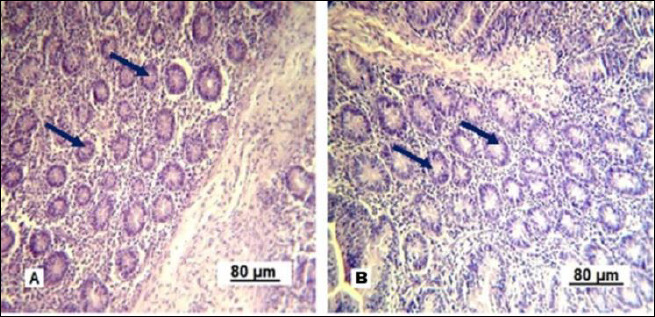
Lieberkühn crypts of the small intestine mucous membrane in replacement gilts. A: control group; B: experimental group. Stained with Boehmer’s hematoxylin and modified Hart’s fuchsia. Corrective optical filter ×2.5. Final magnification ×120. Scale bars = 80 μm.

Under experimental conditions, Lieberkühn crypt secretion is activated (Figure 9). In control gilts, the gland lumina are mainly collapsed and goblet enterocytes are barely visible. In experimental gilts, the glandular lumina are expanded and goblet cells are clearly visible, indicating stimulated secretory activity.

**Figure 10:**
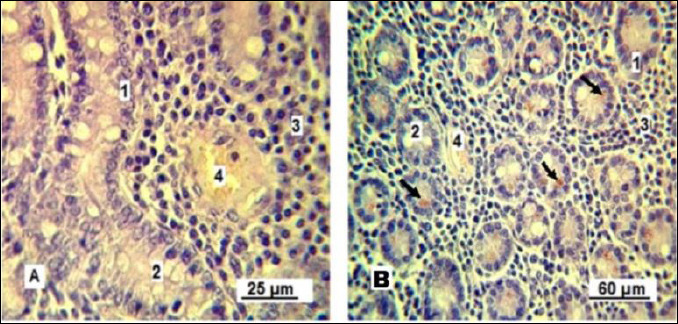
Paneth cells. A: control group; B: experimental group. 1: simple columnar epithelium; 2: goblet cells; 3: lymphoid tissue; 4: blood capillary; 5: Paneth cells (indicated by arrows). Stained with Boehmer’s hematoxylin and modified Hart’s fuchsia. Corrective optical filter ×2.5. Final magnification ×300; ×180. Scale bars: A = 25 μm, B = 60 μm.

An important observation confirming the increased functional of the Lieberkühn crypts is the activation of Paneth cells (Figure 10). These cells appear singly or in small ed groups at the base of glands and are characterized by cytoplasm containing acidophilic granules.

The Paneth cells of control gilts showed weakly eosinophilic cytoplasm, indicating ongoing release of antimicrobial granules needed to sustain intestinal microflora balance. Conversely, Paneth cells in the experimental group contained granules at various stages of development, including intensely stained mature granules that likely serve as a reserve pool.

**Figure 11:**
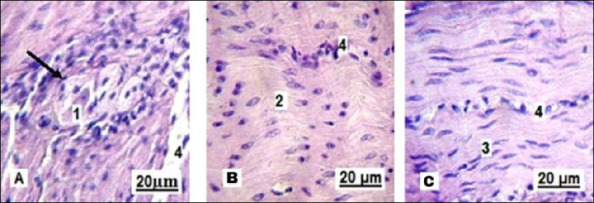
Neuron of the Auerbach’s plexus ganglion: A, indicated by the arrow; and smooth muscle of the circular layer of the small intestine in replacement gilts: B – control group; C – experimental group. 1: activated neuron; 2: relaxed smooth muscle cells; 3: contracted smooth muscle cells; 4: blood capillary. Stained with Boehmer’s hematoxylin and eosin Y stain. Final magnification ×400. Scale bars = 20 μm.

Finally, neuronal activity within the ganglia of Auerbach’s plexus provided insight into intestinal motility (Figure 11). Neurons in the experimental group showed hyperchromatic cytoplasm, indicating increased electrochemical activity, while smooth muscle cells exhibited signs of contraction, suggesting enhanced intestinal peristalsis.

Overall, supplementation with the phytogenic additive “Imunochasnyk” improved productive performance, increased intestinal absorptive capacity, stimulated secretory activity, and enhanced functional activity of the intestinal mucosa in replacement gilts.

## DISCUSSION

### Effects of phytogenic supplementation on growth performance of replacement gilts

This study demonstrates that including the phytogenic feed additive “Imunochasnyk” at a dose of 0.10% in the diet enhances the growth performance and intestinal histomorphology of replacement gilts, while also reducing culling rates and increasing survival rates. The two groups of animals were comparable at 11–12 weeks (around 32 kg), supporting the internal validity of subsequent comparisons. From week 17 onward, the experimental gilts showed higher LW and ADG, and this benefit continued through weeks 22–28, with an improved survival rate (95% vs. 85%).

The observed reduction in FCR in our study is a key indicator of the economic efficiency of the phytogenic additive. Lowering the FCR by approximately 8%–11% significantly decreases the total feed cost needed to reach the target breeding weight of replacement gilts. This improvement is probably connected to the morphological activation of the intestinal mucosa, specifically the increased absorptive surface area of enterocytes, which enables more efficient nutrient extraction from the diet. Enhancements in feed efficiency are crucial for maintaining profitability in antibiotic-free rearing systems in commercial swine production. These performance responses align with those reported in recent literature, which states that phytogenic additives can boost growth by improving gut function, nutrient absorption, and mucosal integrity in pigs [2].

The positive effects of phytogenic feed additives on pig production have been widely documented. Yan *et al*. [36] evaluated a mix of herbal extracts containing buckwheat, thyme, curcuma, black pepper, and ginger in the diet of growing pigs and concluded that it could serve as a natural alternative to AGP because of its beneficial impact on feed intake and weight gain.

Supplementing replacement gilts with “Imunochasnyk” at a level of 0.10% was linked to increased growth rate, better ADG, and higher herd survival. These results support the idea that the tested phytogenic additive has a combined growth-promoting effect in the rearing system of replacement gilts, probably through improvements in gut health and adaptive capacity.

Herbs and plant-derived compounds serve as valuable sources of nutrients and bioactive substances with notable health-promoting properties for humans and animals [37]. Phytogenic feed additives have been shown to primarily enhance the natural defense mechanisms of animals.

### Intestinal epithelial activation and absorptive capacity

Mechanistically, histology supports the idea that increased absorptive capacity drives pig performance parameters. This was confirmed by a 19.6% increase in the average epithelial cell area (44.25 ± 7.43 μm² vs. 36.98 ± 5.92 μm² in Control; p < 0.01) and a significantly higher N:C ratio (0.28 ± 0.04 vs. 0.25 ± 0.03; p < 0.01), indicating enterocyte activation and improved uptake.

Similar improvements in villus architecture (greater villus height and villus:crypt ratio) have been reported in pigs supplemented with phytogenics or botanicals, indicating better absorptive surface and barrier function. Lin *et al*. [38] reported that inclusion of herbal feed additives increases the villus height-to-crypt depth ratio in the ileum, jejunum, and duodenum of pigs, indicating their beneficial role in maintaining intestinal health and improving nutrient digestion and absorption. These morphological improvements are directly associated with enhanced growth performance and overall animal productivity.

Villus thickening and tortuosity, along with increased lymphatic and vascular filling, support enhanced perfusion and absorptive capacity in the experimental group. Recent reviews highlight that villus height/crypt depth metrics remain the gold-standard structural indicators of functional gut health in pigs and correlate with performance outcomes [20]. Although this study did not focus on these traditional linear measurements, the observed cytological changes in the brush-border epithelium strongly indicate improved absorptive ability. In heat stress models, a Capsicum-based phytogenic also improved intestinal morphology and performance, reinforcing the link between phytogenically driven mucosal adaptation and growth [3].

### Modulation of intestinal secretory and immune defense mechanisms

Secretory activity was similarly regulated: crypt lumina expanded and goblet-type enterocytes appeared more frequently in experimental gilts, supporting increased mucus production and better epithelial protection. Similar increases in goblet cell numbers and beneficial crypt–villus restructuring have been reported in phytogenic or probiotic treatments aimed at improving intestinal health in pigs [39].

Beyond goblet cells, Paneth cell activation (granules at various stages of maturation) indicates enhanced innate mucosal defense through the production of lysozyme and antimicrobial peptides, as well as the stabilization of microbial ecology—functions recognized as essential to pig intestinal homeostasis [40]. These findings align with the current study, where activation of goblet and Paneth cells, along with enlargement of crypt lumina, suggests a similar mucosal adaptation pathway influenced by the garlic-based phytogenic additive “Imunochasnyk.”

### Neuromuscular regulation and ENS adaptation

Neuromuscular findings further support improved gastrointestinal function. Changes in myenteric (Auerbach’s) plexus morphology and elongated smooth muscle nuclei in experimental gilts are consistent with enhanced peristalsis. The ENS coordinates motility through the myenteric plexus located between the circular and longitudinal muscle layers; structural and functional adaptations of the ENS are increasingly linked to diet-induced changes in gut transit [41].

In addition to microflora optimization, ENS-linked motility may explain faster nutrient absorption and the observed growth advantages. Overall, our data support that phytogenic supplementation can provide multiple benefits, including structural (villus/crypt and epithelial cell health), secretory (goblet and Paneth cell activity), and neuromuscular (myenteric plexus and peristalsis) effects that contribute to enhanced performance and viability. These results are consistent with recent comprehensive reviews and targeted trials in pigs, reinforcing the scientific basis for using phytogenic additives as antibiotic-free options in gilt rearing [21].

### Role of garlic-based phytogenics in pig nutrition

Recent data show that research has increasingly focused on using garlic (*A. sativum*) as a phytogenic feed additive in both monogastric livestock and poultry, supporting the current findings in replacement gilts. For example, Biswas and Kim [1] demonstrated that including garlic powder in the diet significantly improved growth performance and nutrient digestibility in growing pigs, supporting the idea that garlic-derived bioactive sulfur compounds, such as allicin and alliin, serve as natural growth promoters.

Although the beneficial effects of garlic-based additives have been well documented in piglets and broilers, their application in replacement gilts is of particular strategic importance. Unlike meat-type animals, replacement gilts require a strong intestinal barrier to support long-term metabolic demands and reproductive longevity. Our findings demonstrate that inclusion of 0.10% “Imunochasnyk” supports this developmental goal by enhancing mucosal surface area and cellular activity, which are crucial for the successful transition of gilts into the breeding herd. This study bridges the gap between the general growth promotion observed in other species and the specific requirements for sustainable gilt rearing.

The efficacy of garlic is attributed to its bioactive organosulfur compounds that exhibit antimicrobial, antioxidant, and immunomodulatory properties. Abdelfattah *et al*. [14] highlighted that garlic enhanced the villus height-to-crypt depth ratio, increased the absorptive surface, and reduced oxidative stress under heat or microbial challenge conditions. These morphological enhancements mirror our histomorphometry findings in the small intestine of gilts, including increased brush-border cell area, altered N:C ratio, and enhanced absorptive architecture. We propose that the garlic-based additive “Imunochasnyk” triggered similar epithelial activation, resulting in improved nutrient uptake and subsequent growth gains.

Garlic supplementation has also been shown to enhance digestibility and gut health in pig production. Nikolova [42] summarized multiple trials in which adding garlic powder or extract improved nutrient retention and growth measures and reduced the incidence of diarrhea in pigs. These findings support our study’s observation of decreased culling and an increased survival rate in the experimental group, likely due to improved gut health and immune resilience.

Importantly, garlic phytobiotics can act through multiple converging pathways: enhancing the epithelial absorptive surface, stimulating mucosal secretory and immune cell activity, modifying the composition of the intestinal microbiota, and improving gut motility through neuromuscular activation. Our observations of enhanced villus architecture, activated Paneth cells, and improved peristalsis in gilts align with these mechanisms. Therefore, the overall evidence supports translating garlic-based phytogenic additives from poultry and piglet models to replacement gilts. The consistent benefits of garlic phytobiotics across different species and production stages demonstrate their value as alternatives to antibiotics in intensive livestock systems.

This study shows that even a 0.10% inclusion of a garlic-based product in the replacement gilt diet can lead to significant improvements in performance and survivability. With regulatory and consumer pressure to cut antibiotic use, garlic-based phytobiotics like “Imunochasnyk” offer a practical way to support sustainable and resilient pig production.

### Conceptual mechanism of phytogenic action

In summary, the current results expand the literature by confirming that garlic-derived phytogenic feed additives promote growth and intestinal health in replacement gilts, similar to findings in growing pigs and broilers. They emphasize the cross-species potential of garlic phytobiotic blends and highlight the importance of gut structure–function adaptation as the underlying mechanism. Future research should investigate dose responses, gut microbiota interactions, and long-term reproductive outcomes in sows derived from treated gilts.

**Figure 12:**
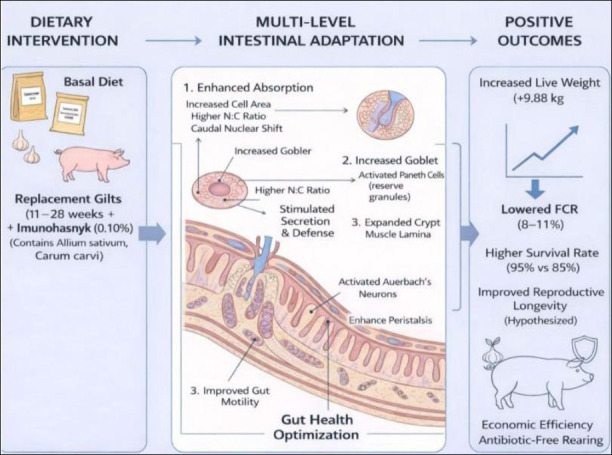
Proposed mechanism of action of the phytogenic additive “Imunochasnyk” on intestinal health and replacement gilt performance parameters. The schematic shows the pathway from dietary intervention to positive economic and physiological outcomes. Dietary intervention: adding 0.10% phytogenic additive to the basal diet of gilts (11–28 weeks). Multi-level intestinal adaptation: (1) improved absorption indicated by increased enterocyte area and N:C ratio; (2) enhanced secretion and defense through activation of goblet and Paneth cells; (3) better motility via activation of Auerbach’s plexus neurons and increased peristalsis. Positive outcomes: notable rise in live weight (+9.88 kg), lower FCR (8%–11%), and improved survival rates (95% vs. 85%), supporting sustainable antibiotic-free rearing.

A conceptual model of the additive’s action is proposed to connect the observed morphofunctional changes with overall performance improvements (Figure 12). This framework shows how the synergistic effects of *A. sativum* and *C. carvi* compounds induce multilevel intestinal adaptation, ultimately leading to improved nutrient utilization and enhanced performance in replacement gilts.

### Limitations

Although the present study provides valuable evidence of the beneficial effects of the phytogenic additive “Imunochasnyk” on growth performance and intestinal morphofunctional characteristics in replacement gilts, several limitations should be considered when interpreting the results.

First, the study was conducted on a single commercial farm, which may limit the generalizability of the findings to other production environments. Differences in farm management practices, pig genetic lines, housing systems, and regional feeding strategies can affect animal performance and intestinal physiology. Therefore, the results of this study may not fully reflect the responses of replacement gilts raised under different climates, production levels, or management systems.

Second, the experiment tested only one inclusion level (0.10%) of the phytogenic additive. While this dose showed positive effects on productive performance and intestinal histology, the lack of multiple dosage groups limits the ability to determine the optimal inclusion rate and to understand the dose–response relationship. Different concentrations of phytogenic compounds may produce various biological effects depending on the animal’s age, physiological status, and diet composition.

Third, the study’s duration was limited to the rearing phase of replacement gilts (11–28 weeks of age). While this developmental stage is crucial for establishing intestinal health and growth potential, the long-term effects of phytogenic supplementation on reproductive performance, sow longevity, and lifetime productivity were not assessed. Since replacement gilts are meant to join the breeding herd, it would be important to determine whether the improvements observed in intestinal morphology and growth lead to better reproductive outcomes, such as age at puberty, litter size, and farrowing performance.

Fourth, the current study mainly examined histological and cytological features of the small intestine to explain the observed performance improvements. While these indicators offer valuable insights into intestinal structure and absorption, the research did not evaluate the composition of intestinal microbiota or microbial metabolic activity. Since phytogenic additives often exert their effects by altering gut microbial populations, the lack of microbiological and metagenomic analyses is a significant limitation to fully understanding their mechanisms of action.

Fifth, biochemical and molecular markers related to intestinal function, oxidative stress, and immune activity were not examined. Measuring digestive enzyme activity, inflammatory cytokines, antioxidant levels, and gene expression related to epithelial turnover or barrier integrity would have provided additional mechanistic evidence supporting the observed histomorphological changes.

Finally, although histological evaluation showed significant differences in epithelial cell shape, goblet cell density, Paneth cell activity, and ENS-related neuromuscular changes, the sample size for microscopic analysis was fairly small. Using more animals and tissue samples could improve the statistical strength and trustworthiness of the histological results.

Although these limitations exist, combining productive performance data with detailed intestinal histology offers a solid foundation for understanding the biological effects of phytogenic supplements in replacement gilts. However, wider multi-farm trials, more mechanistic studies, and long-term reproductive research are necessary to verify the feasibility and sustainability of phytogenic additives in commercial swine production systems.

## CONCLUSION

The present study demonstrated that including the phytogenic feed additive “Imunochasnyk” at 0.10% significantly enhanced both productive performance and intestinal morphofunctional health in replacement gilts during the critical rearing period from 11 to 28 weeks of age. Animals with this supplementation consistently showed higher live weights and ADG from week 17 onward, resulting in a final weight advantage of approximately 9.88 kg compared to the control group. Feed efficiency also improved, indicated by an 8%–11% reduction in FCR, and survival rates increased from 85% in the control group to 95% in the experimental group. Histological and cytological findings supported these performance improvements, showing activation of the intestinal mucosa marked by a 19.6% increase in epithelial cell area, a higher N:C ratio, greater goblet cell density, expanded Lieberkühn crypt lumina, and increased Paneth cell activity. These structural and functional changes collectively suggest enhanced absorption, stronger mucosal defenses, and improved intestinal motility mediated through the myenteric plexus.

From a practical standpoint, these findings show that phytogenic formulations based on *A. sativum* and *C. carvi* can effectively replace AGP in replacement gilt rearing systems. Improved nutrient absorption, enhanced feed efficiency, and lower culling rates directly lead to economic benefits for commercial pig production, especially in environments with restricted antibiotic use. Therefore, the results support the incorporation of garlic-based phytogenic additives into modern swine nutrition plans to enhance herd health, productivity, and sustainability.

A major strength of this study is its integration of production performance data with detailed histological and cytological assessments of the small intestine, including analyses of epithelial cell activity, secretory cell populations, and ENS-related neuromuscular changes. This multi-level approach offers a mechanistic explanation linking intestinal structural and functional adaptation to improved productive performance in replacement gilts.

Future research should build on these findings by examining dose–response relationships of phytogenic additives, their long-term impacts on reproductive performance and sow longevity, and their interactions with intestinal microbiota and metabolic pathways. Additional studies using molecular markers of epithelial turnover, immune signaling, and microbiome composition would help clarify the biological mechanisms underlying the beneficial effects of phytogenic feed additives.

In conclusion, supplementing replacement gilt diets with the garlic-based phytogenic additive “Imunochasnyk” at 0.10% enhances growth performance, benefits intestinal functional capacity, improves feed efficiency, and boosts herd survival. These results strongly support that phytogenic additives serve as a practical and sustainable approach to boosting productivity and intestinal health in antibiotic-free swine production systems.

## DATA AVAILABILITY

The data supporting the findings of this study are available from the corresponding author upon reasonable request.

## AUTHORS’ CONTRIBUTIONS

VL: Conceived and designed the study, coordinated the experimental work, and interpreted the data. NB: Conducted literature searches, performed animal experiments, collected data, prepared histological samples, interpreted performance parameters, performed statistical analysis, validated final data, and visualized the final version of the manuscript. AL: Conducted microscopic and morphometric analyses, prepared visual materials (figures and microphotographs), and contributed to the Results and Discussion sections. IB: Supervised feeding and nutritional schemes, ensured diet formulation, and ensured feed quality control. RF: Provided methodological guidance in experimental design and data validation, and critically reviewed the manuscript for scientific accuracy. VSh: Supervised laboratory analyses and ensured the quality and reproducibility of histological processing. All authors have read and approved the final version of the manuscript.
